# Adaptation and validation of the generative AI dependency scale: evidence from construct validity, reliability and measurement invariance

**DOI:** 10.3389/fpsyg.2026.1908473

**Published:** 2026-08-06

**Authors:** Demet Alkan, Erdem Boduroğlu, Mahmut Sami Yıgıter

**Affiliations:** 1 Department of Educational Measurement and Evaluation, Hacettepe University, Ministry of National Education, Ankara, Türkiye; 2 Department of Educational Measurement and Evaluation, Ereğli Faculty of Education, Necmettin Erbakan University, Konya, Türkiye; 3 Distance Education Application and Research Center, Social Sciences University of Ankara, Ankara, Türkiye

**Keywords:** generative AI dependency, generative artificial intelligence, measurement invariance, reliability, scale adaptation, validity

## Abstract

This study aimed to adapt the Generative AI Dependency Scale into Turkish and to evaluate its psychometric properties in a Turkish sample. The study was conducted with 640 participants. The adaptation process was carried out through translation, back-translation, expert review and cultural evaluation procedures. Construct validity was examined using confirmatory factor analysis (CFA) and both first-order and second-order models were tested. The findings supported the original three-factor structure of the scale, consisting of cognitive preoccupation, negative consequences, and withdrawal. The fit indices indicated that both models demonstrated good fit. Reliability analyses showed that the Turkish form of the scale had acceptable to high internal consistency coefficients for both the subdimensions and the total score. Additional evidence based on the measurement model was obtained through average variance extracted (AVE) and composite reliability (CR) values. Measurement invariance analyses further showed that the scale achieved strict invariance across all examined demographic and AI-related groups. Overall, the findings provide evidence supporting the validity and reliability of the Turkish version of the Generative AI Dependency Scale for assessing dependency on generative artificial intelligence systems.

## Introduction

Recent advances in artificial intelligence have accelerated the development and visibility of generative AI systems capable of producing text, images, audio and other forms of content. As these systems have become more widely discussed and used, scholarly attention has increasingly focused on their role in supporting content generation, decision-related processes and human–AI interaction in professional and educational contexts (Bansal et al., 2024; Chuma and Oliveira, 2023; Hao et al., 2024). At the same time, the rapid expansion of generative AI has prompted growing interest in its broader psychological, behavioral and cognitive implications, suggesting that these tools should be examined not only in terms of utility, but also in terms of their possible effects on users’ thinking and everyday practices (Goh et al., 2025; Klingbeil et al., 2024). Although generative AI systems may increase efficiency and reduce cognitive effort in many contexts, growing reliance on them may also create new risks. Previous studies suggest that excessive reliance on AI may be associated with reduced human agency, weaker critical engagement and increasing dependence in judgment and decision-making processes (Chan, 2020; Klingbeil et al., 2024). Recent work has also drawn attention to concerns such as skill erosion, creative displacement anxiety and the gradual transfer of cognitively demanding or creative tasks to AI systems (Caporusso, 2023; Giray, 2024). In educational contexts, the effects of generative AI on learning and higher-order thinking are still under discussion. However, recent studies indicate that the quality of AI interaction and the quality of generated outputs may shape learning-related outcomes, while systematic reviews point to the need for closer examination of the implications of these technologies for students’ critical thinking.

Such patterns may be interpreted within the broader framework of behavioral addiction. Behavioral addiction refers to the continuation of a behavior despite loss of control and negative consequences in daily functioning (Goodman, 1990; Griffiths, 2005). Models developed for internet and smartphone addiction provide an important starting point for understanding problematic or excessive patterns of technology use (Chen et al., 2003; Kwon et al., 2013; Young, 1998). However, generative AI systems differ from many earlier digital tools in that they function not only as channels for communication or information access, but also as systems that can generate content, support reasoning and partially substitute for human cognitive effort. For this reason, excessive reliance on generative AI may not be fully captured by traditional models of technology addiction alone (Bandi et al., 2023; Goh et al., 2025). Self-Determination Theory also provides a useful framework for interpreting this issue. According to Deci and Ryan (2000), autonomy, competence, and relatedness are basic psychological needs that shape human motivation and behavior. In this context, generative AI tools may initially strengthen perceived competence by helping individuals perform difficult tasks more easily; however, persistent delegation of cognitive effort to AI systems may, over time, weaken autonomous functioning and self-regulation. In other words, while generative AI may serve a supportive role in the short term, excessive and uncontrolled use may gradually erode individuals’ confidence in their own cognitive resources.

Conceptually, generative AI dependency should be distinguished from related but different constructs such as addiction-oriented approaches to digital technology use, problematic online use, habitual technology use, cognitive offloading, and technology acceptance. Technology addiction and addiction-oriented approaches to digital technology use are generally discussed with reference to core addiction-like components such as salience, mood modification, tolerance, withdrawal, conflict, and relapse (Griffiths, 2005), whereas problematic online use, often operationalized through generalized problematic internet use frameworks, emphasizes maladaptive patterns of internet use and their psychological or functional consequences (Caplan, 2010). Habitual technology use refers to repeated and increasingly automatic engagement with a technology and may influence continued use without necessarily indicating dependency or impairment (Limayem et al., 2007). Cognitive offloading describes the use of external tools to reduce the cognitive demands of a task (Risko and Gilbert, 2016). However, unlike conventional forms of cognitive offloading, generative AI tools may support or partially substitute higher-order cognitive processes such as writing, reasoning, summarizing, problem solving, and idea generation. Technology acceptance, in turn, focuses on users’ perceived usefulness, perceived ease of use, and intention to use a technology (Davis, 1989), with more recent AI-specific acceptance models extending this perspective to issues such as privacy concerns and socially responsible AI deployment (Salam et al., 2026a, 2026b). Generative AI dependency overlaps with these constructs but is more specific in that it refers to users’ perceived difficulty in functioning, thinking, or completing tasks without generative AI assistance. Therefore, it reflects not merely frequent use or positive attitudes toward generative AI, but a stronger form of reliance characterized by cognitive preoccupation, negative consequences, and withdrawal-like discomfort when access to generative AI tools is limited or unavailable. These conceptual distinctions are important for interpreting generative AI dependency as a specific form of reliance on generative AI systems rather than as a direct synonym for general technology addiction, problematic internet use, habitual use, cognitive offloading, or technology acceptance.

A further issue concerns the adequacy of existing measurement tools. The current literature on AI-related instruments is largely focused on attitudes, acceptance, anxiety, or general problematic technology use. Yet generative AI differs from conventional digital tools because of its distinctive roles in content generation, decision support, and cognitive offloading. Therefore, more specific instruments are needed to assess dependency on generative AI directly and multidimensionally. In the Turkish context, studies on artificial intelligence have also tended to focus primarily on attitudes, acceptance, or anxiety, whereas instruments designed to assess dependency on generative AI remain limited. Because technology use, trust in cognitive tools and problematic usage patterns may be shaped by cultural context, instruments developed in other cultures may not be fully transferable without adaptation. For this reason, adapting a specific measure of generative AI dependency into Turkish is important both theoretically and practically. Such an instrument may contribute to a more accurate examination of risky patterns of generative AI use among groups that are likely to interact with these tools regularly, such as teachers, academics and office employees.

To situate the present study within the broader literature, examples of instruments related to problematic technology use and generative AI dependency are presented in Table 1.

**Table 1:** Examples of instruments related to problematic technology use and generative AI dependency.

Authors	Instrument	Primary focus	Number of items
Young (1998)	Internet Addiction Test (IAT)	Problematic internet use	20
Chen et al. (2003)	Chinese Internet Addiction Scale	Internet addiction symptoms	26
Kwon et al. (2013)	Smartphone Addiction Scale (SAS)	Problematic smartphone use	33
Schepman and Rodway (2023)	General Attitudes towards AI (GAAIS)	General attitudes and acceptance of AI	20
El-Sayed et al. (2025)	Researchers’ Artificial Intelligence Addiction Scale (RAIAS)	AI addiction/problematic AI use among researchers	22
Goh et al. (2025)	Generative AI Dependency Scale	Dependency on generative AI systems	11

As shown in Table 1, earlier instruments primarily focused on general patterns of problematic technology use, particularly in relation to internet and smartphone use. More recent work, however, has begun to address dependency on generative AI more directly. In this regard, the Generative AI Dependency Scale developed by Goh et al. (2025) is particularly noteworthy because it was designed specifically to assess dependency on generative AI systems. This feature makes the scale especially relevant for adaptation studies focusing on generative AI rather than on broader forms of technology use. In cross-cultural scale adaptation, maintaining the original factor structure is desirable when the adapted form demonstrates linguistic equivalence, construct validity, reliability, and measurement invariance, because it indicates that the same construct can be interpreted consistently across linguistic and cultural contexts. Therefore, the present study evaluated the Turkish form of the Generative AI Dependency Scale through multiple sources of psychometric evidence, including confirmatory factor analysis, second-order factor modeling, reliability coefficients, convergent validity indices, and measurement invariance across demographic and AI-related groups. Establishing such evidence is particularly important because generative AI dependency is an emerging construct that may be shaped by cultural context, professional use patterns, and the degree to which generative AI tools are embedded in daily cognitive and work-related routines. Accordingly, the present study aimed to adapt the Generative AI Dependency Scale into Turkish and to examine its psychometric properties in a Turkish sample. More specifically, the study sought to evaluate the linguistic and cultural suitability of the scale, test its original three-factor structure through confirmatory factor analysis, examine its reliability, and assess measurement invariance across different demographic and AI-related groups.

## Method

### Research design

The present study was designed as a scale adaptation and psychometric validation study. The aim was to adapt the Generative AI Dependency Scale into Turkish and to examine its psychometric properties in a Turkish sample. Within this scope, the study focused on evaluating the linguistic and cultural suitability of the scale, confirming its original factor structure, examining its reliability and testing measurement invariance across different demographic and AI-related variables.

### Participants

The study sample consisted of 640 individuals who voluntarily participated in the study through an online survey. The sample size was considered adequate for the planned psychometric analyses, including confirmatory factor analysis and measurement invariance testing (Hair et al., 2022a, 2022b; Kline, 2023). Furthermore, for large populations, a minimum sample size of 384 is recommended at a 95% confidence level with a 5% margin of error (Krejcie and Morgan, 1970). Accordingly, the sample of 640 participants exceeded the recommended minimum sample size. The demographic characteristics of the participants and their AI-related characteristics are presented in Table 2.

**Table 2:** Demographic characteristics of the participants and AI-related variables.

Variable	Category	*n*	%
Gender	Female	363	56.7
	Male	277	43.3
Educational level	Bachelor’s degree or below	283	44.2
	Master’s degree	224	35.0
	Doctoral degree	133	20.8
AI knowledge level	Low	196	30.6
	Moderate	405	63.3
	Advanced	39	6.1
AI use status	User	592	92.5
	Non-user	48	7.5
Duration of AI use	Less than 6 months	122	19.1
	6–12 months	169	26.4
	1–2 years	250	39.1
	More than 2 years	99	15.5
Interaction complexity	Simple queries/basic tasks	215	33.6
	Querying and content editing	259	40.5
	Complex/advanced multi-step tasks	166	25.9
Subscription status	Free services only	431	67.3
	Paid access	209	32.7
Age	Up to 30 years	274	42.8
	31–40 years	220	34.4
	40+ years	146	22.8

As shown in Table 2, the sample varied across demographic characteristics and AI-related background variables. Most participants reported using AI tools (92.5%), whereas 7.5% identified themselves as non-users. Regarding self-reported AI knowledge, most participants reported a moderate level of knowledge (63.3%), followed by low (30.6%), and advanced (6.1%) levels. In terms of duration of AI use, 39.1% had used AI tools for 1–2 years, 26.4% for 6–12 months, 19.1% for less than 6 months, and 15.5% for more than 2 years. Participants also differed in interaction complexity: 33.6% reported simple or basic use, 40.5% reported querying or content-editing use, and 25.9% reported complex or multi-step use. Finally, 67.3% used free AI tools, whereas 32.7% reported using paid subscriptions. Overall, these characteristics provide a clearer contextual profile of the validation sample and indicate that most participants had direct experience with AI tools, while also varying in AI knowledge, duration of use, and interaction complexity.

### Instrument

The Generative AI Dependency Scale was developed by Goh et al. (2025) and published in *Computers in Human Behavior Reports*. The scale was designed to assess individuals’ dependency on generative artificial intelligence systems within a multidimensional framework. Its development was informed by two main theoretical frameworks: behavioral addiction theory and Self-Determination Theory. Within the behavioral addiction framework, the construct was approached in relation to core components such as salience, compulsive use, negative consequences and withdrawal. From the perspective of Self-Determination Theory, dependency on generative AI was conceptualized as a potential compensatory mechanism associated with unmet psychological needs such as autonomy, competence and relatedness.

The validity and reliability studies of the original scale were conducted through six studies involving a total of 1,333 participants from the United States and Singapore. During the scale development process, an initial pool of 30 items was generated based on the technology addiction literature, including studies on internet and smartphone addiction and the items were then revised to reflect the distinctive characteristics of generative AI systems, such as content generation and decision support. In this process, some dimensions commonly discussed in traditional addiction models were excluded from the final structure. In particular, the tolerance dimension was not retained because increasing efficiency in the use of generative AI does not necessarily reflect the same pattern observed in conventional addiction frameworks. Similarly, the mood regulation dimension was removed because of its substantial conceptual overlap with negative consequences.

Following exploratory and confirmatory factor analyses, the scale reached a stable structure consisting of 11 items and three subdimensions: cognitive preoccupation, negative consequences and withdrawal. Cognitive preoccupation refers to persistent mental focus on and the urge to use generative AI systems. Negative consequences refer to the adverse effects of excessive use on performance and self-efficacy. Withdrawal refers to feelings of discomfort or distress when access to generative AI systems is limited. In the original study, the scale demonstrated strong psychometric properties. Internal consistency coefficients ranged between 0.92 and 0.93 and the test–retest reliability coefficient over a one-week interval was reported as 0.87. In addition, the scale showed evidence of convergent and discriminant validity and measurement invariance was supported across gender and cultural groups (Goh et al., 2025). The scale is a five-point Likert-type instrument ranging from 1 (*strongly disagree*) to 5 (*strongly agree*). Higher scores indicate higher levels of dependency on generative AI systems.

### Translation and cultural adaptation process

The adaptation of the Generative AI Dependency Scale into Turkish was carried out in line with commonly recommended procedures for test adaptation and cross-cultural translation. In this process, the aim was to minimize unnecessary changes to the original scale while ensuring its linguistic clarity, conceptual equivalence and cultural appropriateness in the target context. Accordingly, the Turkish adaptation process was conducted on the basis of the linguistic and cultural adaptation steps proposed by Hambleton and Patsula (1998) and the standards outlined by the International Test Commission (2017).

At the initial stage of the adaptation process, the conceptual framework, structure and measured components of the original form of the Generative AI Dependency Scale were examined in detail. To preserve conceptual equivalence, the wording of the items was reviewed carefully with attention to experiential and contextual relevance and possible differences in meaning across cultures were taken into consideration. In addition, the suitability of the original construct for the Turkish context was evaluated by considering both the purpose and the content of the scale. Throughout this process, particular attention was paid to preserving the conceptual structure of the scale and to ensuring the cultural and contextual appropriateness of the translated items.

The linguistic adaptation process involved translation of the original scale, evaluation of the translated versions and preparation of a trial form for comparison with the original instrument. In the present study, the scale was independently translated into Turkish by three bilingual experts proficient in both Turkish and English. These independent translations were compared and a preliminary Turkish form was created by evaluating the items in terms of semantic equivalence, linguistic clarity and cultural suitability. This preliminary version was then back-translated into English by three independent bilingual experts who were not involved in the initial translation process. The back-translated version was compared with the original form in order to identify potential discrepancies in meaning and wording. Following this comparison, items were revised where necessary to improve clarity and reduce possible linguistic inconsistencies. Finally, the Turkish form was reviewed by three faculty members from relevant fields, namely Computer Education and Instructional Technology, Turkish Language and Educational Measurement and Evaluation. Based on expert feedback, minor revisions were made to improve the comprehensibility and linguistic appropriateness of the items. At the end of this process, the Turkish version of the scale was considered suitable for use in the main study.

### Data collection procedure

The data for this study were collected online using a structured digital survey form. Participation was entirely voluntary and respondents were informed about the purpose of the study before completing the survey. Prior to data collection, participants were presented with an informed consent statement explaining the aim of the research, the approximate completion time, the voluntary nature of participation and the confidentiality of their responses. Only those who agreed to participate were allowed to proceed to the survey items. Written informed consent was obtained from all participants prior to their participation in the study. Participants were informed about the purpose of the research, the voluntary nature of participation, and the confidentiality of their responses.

The online format enabled efficient access to participants from different professional backgrounds and facilitated the collection of a sufficiently large sample for the adaptation and validation analyses. The survey form consisted of two main sections. The first section included demographic questions and items related to AI use, whereas the second section contained the Turkish form of the Generative AI Dependency Scale. Data collection continued until the target sample size required for the planned psychometric analyses was reached. At the end of the process, responses from 640 participants were retained for the final analyses.

### Data analysis

Because the present study was designed as a scale adaptation and psychometric validation study, the analyses focused on testing the hypothesized factor structure of the adapted form rather than exploring a new structure. Prior to the main analyses, the dataset was screened for missing values. Item-level missing data rates were very low, ranging from 0.00 to 0.78% across the scale items. Given this negligible level of missingness, the Expectation–Maximization (EM) algorithm was used to impute missing responses (Dempster et al., 1977), thereby preserving the full sample for subsequent psychometric analyses. Following the missing data procedure, the distributional characteristics of the scale scores were examined using skewness and kurtosis coefficients. The skewness values for the subdimensions and the total score ranged from 0.64 to 1.25, whereas kurtosis values ranged from −0.13 to 1.75. Since these values remained within generally acceptable limits for multivariate analyses, the data were considered to show an acceptable distributional pattern (Tabachnick and Fidell, 2013).

To examine construct validity, confirmatory factor analysis (CFA) was conducted to test the fit of the original three-factor structure. In addition to the first-order CFA model (Model I), a second-order CFA model (Model II) was also tested to evaluate whether the three first-order dimensions loaded onto a higher-order construct representing overall generative AI dependency. Model fit was evaluated using multiple goodness-of-fit indices, including *χ*^2^/df, CFI, TLI, RMSEA, SRMR, GFI, and AGFI. The following conventional cut-off values were used to interpret model fit: *χ*^2^/df < 5, CFI ≥ 0.90, TLI ≥ 0.90, RMSEA ≤ 0.08, and SRMR ≤ 0.08 (Hu and Bentler, 1999; Kline, 2023). To provide evidence of convergent validity based on the measurement model and internal consistency, average variance extracted (AVE), composite reliability (CR), Cronbach’s alpha and McDonald’s omega coefficients were calculated for each subdimension and for the total scale. In line with the literature, AVE values above 0.50 were considered evidence of convergent validity, whereas CR, alpha and omega values above 0.70 were interpreted as indicating acceptable reliability (Hair et al., 2022a, 2022b; Raykov et al., 2016). In the final stage, measurement invariance was examined across the demographic and AI-related variables included in the study. Multi-group confirmatory factor analysis (MG-CFA) was performed by sequentially testing configural, metric, scalar and strict invariance models. Invariance decisions were based on changes in comparative fit index (ΔCFI) values across adjacent hierarchical models. Following the recommendation of Cheung and Rensvold (2002), a ΔCFI value smaller than 0.01 was accepted as evidence of measurement invariance across levels. All analyses were conducted in R using scripts prepared by the researchers. Missing data imputation was performed using EM-based procedures, whereas CFA and multi-group CFA analyses were conducted with the lavaan package (Rosseel, 2012). Reliability estimates and descriptive analyses were supported by the psych package (Revelle, 2015) and CFA path diagrams were visualized using semPlot (Epskamp, 2015).

## Results

### Construct validity

To examine the construct validity of the Generative AI Dependency Scale, a confirmatory factor analysis (CFA) was conducted to test whether the hypothesized measurement model was supported by the observed data. CFA is one of the most widely used approaches for evaluating construct validity, as it assesses the extent to which the observed items adequately represent the proposed latent structure of the scale. The model fit indices presented in Table 3 were used to determine the degree of model fit to the empirical data.

**Table 3:** CFA model fit indices.

Scale	Reference value	Model I	Model II
*χ* ^2^	–	112.352	120.141
df	–	41	41
*p* value	>0.05	0.000	0.000
*χ*^2^/df	<5	2.740	2.930
RMSEA	<0.07	0.053	0.055
SRMR	<0.07	0.025	0.026
GFI	>0.90	0.999	0.999
AGFI	>0.90	0.998	0.998
NFI	>0.90	0.993	0.993
CFI	>0.92	0.996	0.995
TLI	>0.90	0.994	0.994
IFI	>0.90	0.996	0.995
RFI	>0.90	0.991	0.990

Model I: three factor CFA model, Model II: second level CFA model.

**Figure 1:**
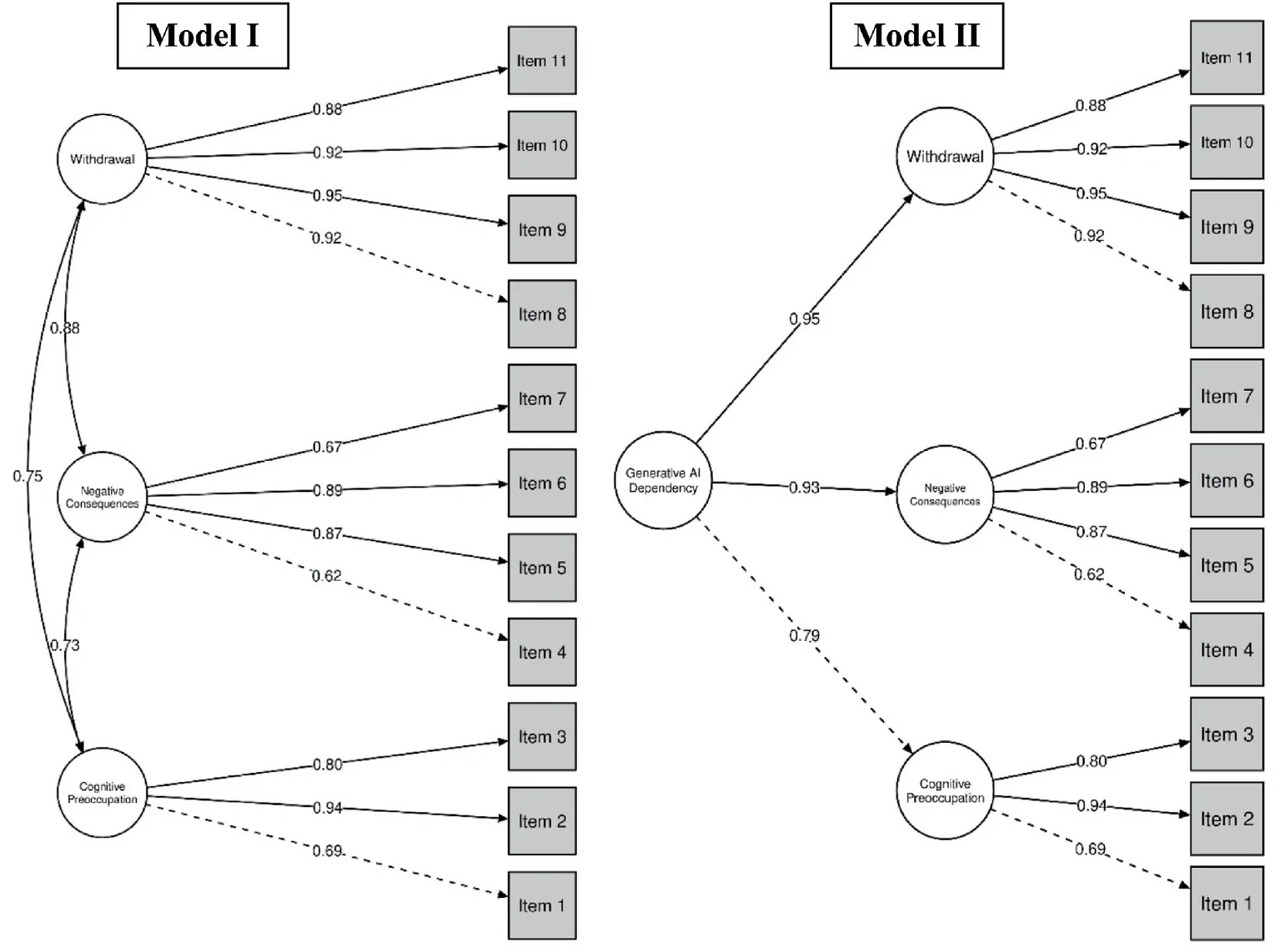
CFA path diagram for Model I and Model II. Model I: three factor CFA model, Model II: second level CFA model.

As shown in Figure 1, the confirmatory factor analysis (CFA) results indicated that Model I and Model II of the Generative AI Dependency Scale demonstrated a good fit to the data. The chi-square value was statistically significant (*χ*^2^ = 112.352, df = 41, *p* < 0.001), which is common in large samples. The *χ*^2^/df ratio was 2.740, indicating a good level of fit. The RMSEA value (0.053) and SRMR value (0.025) were both below recommended thresholds, supporting good model fit. Incremental fit indices were also excellent, with CFI = 0.996, TLI = 0.994, NFI = 0.993, IFI = 0.996, and RFI = 0.991. In addition, GFI (0.999) and AGFI (0.998) values were well above acceptable levels. In addition, the second order CFA model (Model II) also demonstrated an excellent model fit, indicating that the three first-order factors adequately load onto a higher order general Generative AI Dependency construct. Overall, these findings indicate that both Model I and Model II provide an acceptable to good representation of the data and supports the construct validity of the Generative AI Dependency Scale.

The standardized factor loadings indicated that the items under the cognitive preoccupation factor ranged between 0.686 and 0.937, the items under the negative consequences factor ranged between 0.624 and 0.894 and the items under the withdrawal factor ranged between 0.877 and 0.951. Overall, all factor loadings were above 0.60, indicating strong relationships between the items and their respective latent constructs.

### Model-based convergent validity and reliability

Table 4 presents the factor loadings, corrected item-total correlations, average variance extracted (AVE), composite reliability (CR), explained variance and reliability coefficients of the 11-item Generative AI Dependency Scale. These results were used to evaluate convergent validity and internal consistency.

**Table 4:** AVE, reliability and explained variance percentages of the Generative AI dependency scale.

Factor	Items	Factor loadings	Item-total	AVE	CR	Explained variance (%)	Alpha	Omega
Cognitive preoccupation	Item 1	0.686	0.57	0.663	0.805	66.3	0.78	0.79
	Item 2	0.937	0.67					
	Item 3	0.801	0.63					
Negative consequences	Item 4	0.624	0.50	0.598	0.819	59.8	0.79	0.84
	Item 5	0.872	0.66					
	Item 6	0.894	0.69					
	Item 7	0.667	0.56					
Withdrawal	Item 8	0.921	0.81	0.842	0.919	84.2	0.92	0.94
	Item 9	0.951	0.87					
	Item 10	0.919	0.83					
	Item 11	0.877	0.76					
Generative AI dependency							0.91	0.93

The model-based convergent validity results of the Generative AI Dependency Scale indicate that all subdimensions met the recommended criteria. The AVE values were 0.663 for cognitive preoccupation, 0.598 for negative consequences and 0.842 for withdrawal, all exceeding the 0.50 threshold, thus supporting convergent validity evidence within the measurement model. The Cronbach’s alpha coefficients were 0.78, 0.79 and 0.92 for cognitive preoccupation, negative consequences and withdrawal, respectively, while the overall scale alpha was 0.91. Similarly, McDonald’s omega coefficients were 0.79, 0.84 and 0.94 for the three sub-dimensions, with an overall omega of 0.93. These values indicate acceptable to high internal consistency, with particularly strong reliability for the withdrawal dimension. The composite reliability (CR) values were 0.805, 0.819 and 0.919, all above the recommended 0.70 level, further confirming the reliability of the constructs. Factor loadings ranged between 0.624 and 0.951, indicating strong item-factor relationships across all dimensions. Additionally, corrected item-total correlations ranged between 0.50 and 0.87, which are within acceptable limits. The explained variance values were 66.3, 59.8, and 84.2% for the three sub-dimensions, all of which are considered sufficient in social science research. Overall, these findings provide evidence of convergent validity based on the measurement model and indicate high reliability for the Generative AI Dependency Scale, supporting its use for assessing dependency on generative AI systems within the scope of the psychometric evidence examined in the present study.

### Translation and linguistic review

Linguistic equivalence of the scale was supported through a systematic process involving translation, back-translation and expert review. In the first stage, the original English form was independently translated into Turkish by three bilingual experts proficient in both languages and specialized in relevant fields, such as Computer Education and Instructional Technology and Educational Measurement and Evaluation. These independent translations were compared for consistency and a synthesized draft version was created.

In the second stage, this preliminary Turkish form was back-translated into English by three different independent language experts who were blind to the original instrument. The back-translated items were compared with the original scale to evaluate semantic and conceptual consistency and the comparisons suggested that the items largely preserved their intended meanings. In the final stage, the Turkish form was reviewed by three faculty members in terms of linguistic clarity and content appropriateness. Based on their feedback, minor revisions were made to improve comprehensibility and cultural suitability. Overall, these procedures supported the linguistic equivalence of the Turkish form with the original instrument.

### Measurement invariance

Measurement invariance was examined through multi-group confirmatory factor analysis (MG-CFA) across demographic and AI-related variables by sequentially testing configural, metric, scalar and strict models (see Table 5). The decision regarding invariance at each successive step was based on changes in the CFI statistic (∆CFI). Following the criterion proposed by Cheung and Rensvold (2002), measurement invariance was considered supported when the decrease in CFI between adjacent models remained below 0.01.

**Table 5:** Measurement invariance across demographic and AI-related variables.

Demographic variable	Stage	*χ* ^2^	df	*χ*^2^/df	𝑅𝑀𝑆𝐸𝐴	𝑆𝑅𝑀𝑅	GFI	AGFI	TLI	CFI	Δ CFI
Gender	Configural	127,501	82	1.56	0.042	0.030	0.999	0.998	0.996	0.997	–
	Metric	95,680	92	1.04	0.011	0.032	0.999	0.998	0.999	0.999	0.002
	Scalar	106,300	99	1.07	0.015	0.033	0.999	0.998	0.999	0.999	0.000
	Strict	120,833	110	1.10	0.018	0.037	0.999	0.998	0.998	0.998	0.001
Age level	Configural	153,432	123	1.25	0.034	0.033	0.999	0.998	0.997	0.998	–
	Metric	177,457	143	1.24	0.034	0.054	0.997	0.996	0.991	0.992	0.006
	Scalar	197,739	157	1.26	0.035	0.056	0.997	0.995	0.991	0.991	0.001
	Strict	229,275	179	1.28	0.036	0.064	0.996	0.995	0.990	0.989	0.002
Education level	Configural	169,292	123	1.38	0.042	0.035	0.999	0.998	1.000	1.000	–
	Metric	146,934	143	1.03	0.011	0.043	0.998	0.997	1.000	1.000	0.000
	Scalar	161,929	157	1.03	0.012	0.045	0.998	0.997	1.000	1.000	0.000
	Strict	181,274	179	1.01	0.008	0.048	0.998	0.997	1.000	1.000	0.000
AI use status	Configural	125,569	82	1.53	0.041	0.027	0.999	0.999	0.997	0.998	–
	Metric	127,976	92	1.39	0.035	0.035	0.998	0.997	0.992	0.993	0.005
	Scalar	144,986	99	1.47	0.038	0.037	0.998	0.997	0.990	0.991	0.002
	Strict	159,484	110	1.45	0.038	0.040	0.997	0.996	0.989	0.989	0.002
AI experience duration	Configural	209,949	164	1.28	0.042	0.037	0.999	0.998	1.000	1.000	–
	Metric	200,135	194	1.03	0.014	0.052	0.998	0.996	1.000	1.000	0.000
	Scalar	240,428	215	1.12	0.027	0.057	0.997	0.996	1.000	1.000	0.000
	Strict	291,525	248	1.18	0.033	0.069	0.996	0.995	1.000	1.000	0.000
AI interaction complexity	Configural	156,371	123	1.27	0.036	0.033	0.999	0.998	1.000	1.000	–
	Metric	148,768	143	1.04	0.014	0.044	0.998	0.997	1.000	1.000	0.000
	Scalar	187,083	157	1.19	0.030	0.050	0.998	0.996	1.000	1.000	0.000
	Strict	223,522	179	1.25	0.034	0.060	0.997	0.996	1.000	1.000	0.000
AI payment status	Configural	118,901	82	1.45	0.038	0.029	0.999	0.999	0.997	0.998	–
	Metric	110,479	92	1.20	0.025	0.038	0.999	0.998	0.997	0.997	0.001
	Scalar	127,343	99	1.29	0.030	0.040	0.998	0.998	0.996	0.996	0.001
	Strict	136,906	110	1.25	0.028	0.043	0.998	0.998	0.996	0.996	0.000
AI knowledge level	Configural	151,090	123	1.23	0.033	0.031	0.999	0.998	0.998	0.998	–
	Metric	156,842	143	1.10	0.021	0.042	0.998	0.997	0.997	0.998	0.001
	Scalar	177,161	157	1.13	0.025	0.044	0.998	0.997	0.996	0.997	0.001
	Strict	203,342	179	1.14	0.025	0.051	0.997	0.997	0.996	0.996	0.001

The multi-group CFA results provide evidence for the robustness of the scale across demographic and AI-related subgroups. Across all examined variables, including gender, age level, education level, AI use status, AI experience duration, AI interaction complexity, AI payment status and AI knowledge level, the changes in CFI remained below the recommended threshold at each successive stage. These findings support strict measurement invariance across the groups examined. In practical terms, these results indicate that the factor loadings, intercepts and residual variances were comparable across groups. Accordingly, the Turkish version of the Generative AI Dependency Scale appears to function similarly across different demographic and AI-related categories, suggesting that observed group differences are less likely to be attributable to measurement bias. This supports the use of the Turkish form in future comparative studies examining generative AI dependency across demographic characteristics and AI-related usage profiles. However, such comparisons should be interpreted within the group categories and sampling conditions examined in the present study.

### How to score the scale

The Generative AI Dependency Scale consists of 11 items in total and is structured as a second-order measurement model with three first-order dimensions. The cognitive preoccupation dimension includes 3 items, while the negative consequences and withdrawal dimensions each consist of 4 items. In addition, these three dimensions load onto a higher-order latent construct representing overall generative AI dependency. The finalized items and their dimension assignments are presented in the Appendix section for reference.

The scores obtained from the items may be used both at the subdimension level and as an overall total score. Specifically, the items within each dimension can be summed to obtain separate subscale scores for cognitive preoccupation, negative consequences and withdrawal. In addition, because the higher-order CFA results support a general dependency construct, all 11 items may also be summed to obtain a single overall Generative AI Dependency score. Since all items are scored in the same direction, no reverse coding is required. Thus, higher total scores indicate greater dependency on generative AI, whereas lower scores reflect lower levels of dependency. These findings suggest that the scale may be used both for multidimensional profiling and for deriving a global dependency score.

## Discussion and conclusion

The present study adapted the Generative AI Dependency Scale into Turkish and examined its psychometric properties in a Turkish sample. Overall, the findings indicate that the Turkish form of the scale provides a valid and reliable instrument for assessing individual differences in dependency on generative AI systems. Confirmatory factor analysis supported the original three-factor structure consisting of cognitive preoccupation, negative consequences and withdrawal, which is consistent with the original conceptualization of the scale proposed by Goh et al. (2025). In addition, the second-order CFA model also demonstrated acceptable fit, suggesting that the three subdimensions may be interpreted not only separately but also as indicators of a broader construct representing overall generative AI dependency. Taken together, these findings suggest that the conceptual structure of the original scale was preserved in the Turkish adaptation.

Internal consistency coefficients for both the total scale and the subdimensions were within acceptable to high ranges, and the AVE and CR values provided evidence of convergent validity based on the measurement model and composite reliability. These results indicate that the items function coherently within their intended dimensions and that the adapted form yields consistent measurements. In this respect, the Turkish version appears to retain the psychometric strength reported in the original study (Goh et al., 2025). In addition, measurement invariance analyses supported strict invariance across all examined demographic and AI-related variables, including gender, age level, education level, AI use status, AI experience duration, AI interaction complexity, AI payment status and AI knowledge level. These findings indicate that the scale operates similarly across different participant groups and allows meaningful cross-group comparisons in future research.

A finding that warrants specific interpretation is the particularly high internal consistency of the withdrawal dimension (*α* = 0.92, *ω* = 0.94), which substantially exceeded the coefficients obtained for the cognitive preoccupation (*α* = 0.78) and negative consequences (*α* = 0.79) dimensions. This pattern may reflect the psychometric nature of withdrawal as a construct. Withdrawal-related items tend to capture a narrowly bounded, affectively concrete experience namely, distress and discomfort arising from restricted access to generative AI which renders the subscale inherently more homogeneous than dimensions characterized by broader cognitive or behavioral content (Boyle, 1991; Kline, 1986). From a measurement standpoint, narrower constructs with tightly overlapping content tend to yield higher alpha values, not necessarily because they are psychometrically superior, but because their items converge on a more circumscribed psychological state (Boyle, 1991). This interpretive point is important: the high alpha of the withdrawal subscale may reflect genuine construct homogeneity rather than item redundancy, given that the AVE value for this dimension was 0.842 and factor loadings ranged from 0.877 to 0.951, indicating strong and discriminant item-factor relationships. This pattern is broadly consistent with the broader technology addiction literature. For instance, the Smartphone Addiction Scale (Kwon et al., 2013) and its short version, as well as AI-focused scales developed for other populations (El-Sayed et al., 2025), have similarly reported that withdrawal-related subscales sometimes yield relatively high internal consistency coefficients, while other dimensions capturing more diffuse behavioral or cognitive phenomena show more moderate values. In the present Turkish sample, the high withdrawal reliability may also carry a context-specific interpretation. Given that the sample was composed entirely of professionals who actively use generative AI tools in their work with 92.5% reporting active use and 39.1% reporting one to 2 years of sustained experience the withdrawal subscale may have captured a psychologically salient and highly consistent experiential state. For individuals whose professional routines have become substantially organized around generative AI tools, the prospect of losing access to these tools may be experienced in a particularly uniform and acute manner, producing high inter-item agreement within this subscale. This interpretation, while plausible, is inferential in nature and should be examined more directly in future studies using experimental or longitudinal designs.

From a theoretical perspective, the findings support the view that dependency on generative AI can be conceptualized as a multidimensional construct rather than as a simple indicator of frequent technology use. The confirmation of the dimensions of cognitive preoccupation, negative consequences and withdrawal suggests that dependency on generative AI shares certain features with broader behavioral addiction frameworks while also reflecting the distinctive nature of generative AI systems as tools for content generation, reasoning support, and cognitive offloading (Goodman, 1990; Griffiths, 2005; Goh et al., 2025). In addition, the findings may be considered in relation to Self-Determination Theory. From this perspective, frequent and excessive reliance on generative AI may be associated with changes in how individuals experience competence and autonomy when performing cognitively demanding tasks (Deci and Ryan, 2000). Although the present study did not directly test these psychological mechanisms, the confirmation of the scale structure in the Turkish sample suggests that this theoretical perspective remains relevant for understanding dependency on generative AI in different cultural contexts.

As this study is a cross-cultural adaptation, the findings should be interpreted not only in relation to the psychometric evidence but also in relation to the broader context in which generative AI is adopted and used in Turkey. In this regard, Turkey has pursued AI development under a National Artificial Intelligence Strategy (2021–2025) accompanied by a 2024–2025 action plan covering more than 70 measures, reflecting a state-led commitment to rapid digital transformation across educational, academic and professional sectors (Digital Transformation Office of Turkey, 2021). At the individual level, this momentum is reflected in recent official data: according to the Turkish Statistical Institute’s first national AI statistics report, 19.2% of the Turkish population reported using generative AI tools in 2025, with this figure reaching 36.1% among higher education graduates (TurkStat, 2025). Consistent with this pattern, 92.5% of the present sample reported active use of generative AI tools and a notable majority described using these tools for content editing and querying tasks that are directly integrated into their professional workflows. This occupational embeddedness of generative AI use may have important implications for the dependency-related findings observed here. When generative AI tools are adopted rapidly and become integrated into daily professional practice, users’ perceptions of dependency may be shaped by the extent to which these tools become embedded in task completion, productivity expectations and self-regulation practices (Salam et al., 2026a, 2026b). This interpretation is consistent with theoretical accounts suggesting that cognitive offloading onto AI systems may initially enhance perceived competence but gradually erode autonomous functioning and self-regulatory capacity over time (Deci and Ryan, 2000; Gerlich, 2025). Furthermore, recent empirical work indicates that greater AI dependency is associated with reductions in critical thinking and increased cognitive fatigue, particularly when AI tools are used as substitutes for active cognitive engagement rather than as supplements to it (Gerlich, 2025). In the Turkish professional context, where performance pressures in educational and administrative settings are high and AI tools offer significant cognitive relief, the risk of transitioning from assisted performance to dependency may be particularly relevant. The present study’s adapted scale may therefore serve not only as a research instrument but also as a practical tool for identifying at-risk use patterns in Turkish professional settings undergoing rapid AI integration. These contextual interpretations are necessarily post-hoc in nature; therefore, future cross-cultural studies should examine how occupational, institutional and cultural factors shape the meanings of cognitive preoccupation, negative consequences and withdrawal-like discomfort in generative AI dependency.

The study also has practical implications. As generative AI tools become increasingly visible in educational, academic, and professional settings, the availability of a Turkish instrument may facilitate more systematic research on problematic or excessive forms of use. The adapted scale may be useful in studies examining how dependency on generative AI relates to variables such as self-efficacy, academic or work-related outcomes and technology-use patterns. In applied settings, the scale may also provide a structured means of assessing levels of dependency on generative AI among groups that are likely to interact with such tools regularly. In conclusion, the findings of the present study provide evidence supporting the validity and reliability of the Turkish version of the Generative AI Dependency Scale for assessing dependency on generative AI systems within the scope of the psychometric evidence examined in this study and offer a useful tool for future research in Turkish-speaking contexts.

## Limitations and future directions

The present findings should be interpreted in light of several limitations. Data were collected using self-report measures in an online format, which may increase the risk of response bias. The study employed a cross-sectional design, which does not allow for causal or temporal inferences; therefore, longitudinal validity, predictive validity, and temporal stability of generative AI dependency could not be examined. In addition, although convergent validity was assessed using measurement-model-based evidence, no external validation measures were included; thus, criterion-related, predictive, and discriminant validity could not be tested.

Future research should examine the Turkish form alongside related constructs such as generative AI addiction, problematic AI use, AI trust, AI literacy, technology anxiety, and cognitive offloading, and evaluate whether scale scores predict relevant academic, occupational, and behavioral outcomes. The scale should also be tested in diverse populations, including university students, adolescents, and different occupational groups, to assess the stability of its psychometric properties across age groups and social contexts. Further studies may explore relationships between generative AI dependency and variables such as self-efficacy, burnout, cognitive offloading, academic and work performance, and technology-related attitudes. Longitudinal designs are particularly needed to examine temporal stability and predictive validity. Finally, cross-cultural comparisons and studies establishing potential cut-off scores or risk profiles would further enhance the applied interpretation of generative AI dependency.

## Data Availability

The data that support the findings of this study are openly available in the Open Science Framework (OSF) at: https://osf.io/nkdtu, without restriction.

## References

[ref1] Bandi A. Adapa P. V. S. R. Kuchi Y. E. V. P. K. (2023). The power of generative AI: a review of requirements, models, input–output formats, evaluation metrics, and challenges. Fut. Int. 15:260. doi: 10.3390/fi15080260

[ref2] Bansal G. Nawal A. Chamola V. Herencsar N. (2024). Revolutionizing visuals: the role of generative AI in modern image generation. ACM Trans. Multim. Comput. Commun. Appl. 20, 1–22. doi: 10.1145/3689641

[ref3] Boyle G. J. (1991). Does item homogeneity indicate internal consistency or item redundancy in psychometric scales. Pers. Individ. Differ. 12, 291–294. doi: 10.1016/0191-8869(91)90115-R

[ref4] Caplan S. E. (2010). Theory and measurement of generalized problematic internet use: a two-step approach. Comput. Hum. Behav. 26, 1089–1097. doi: 10.1016/j.chb.2010.03.012

[ref5] Caporusso N. (2023). Generative artificial intelligence and the emergence of creative displacement anxiety: review. Res. Dir. Psych. Behav. 3:10795. doi: 10.53520/rdpb2023.10795

[ref6] Chan B. (2020). The rise of artificial intelligence and the crisis of moral passivity. AI Soc. 35, 991–993. doi: 10.1007/s00146-020-00953-9

[ref7] Chen S.-H. Weng L.-J. Su Y.-J. Wu H.-M. Yang P.-F. (2003). Development of a Chinese internet addiction scale and its psychometric study. Chin. J. Psychol. 45, 279–294. doi: 10.6129/CJP.2003.4503.05

[ref8] Cheung G. W. Rensvold R. B. (2002). Evaluating goodness-of-fit indexes for testing measurement invariance. Struct. Equ. Model. Multidiscip. J. 9, 233–255. doi: 10.1207/S15328007SEM0902_5

[ref9] Chuma E. L. Oliveira G. G. D. (2023). Generative AI for business decision-making: a case of ChatGPT. Manage. Sci. Busi. Deci. 3, 5–11. doi: 10.52812/msbd.63

[ref10] Davis F. D. (1989). Perceived usefulness, perceived ease of use, and user acceptance of information technology. MIS Q. 13, 319–340. doi: 10.2307/249008

[ref11] Deci E. L. Ryan R. M. (2000). The “what” and “why” of goal pursuits: human needs and the self-determination of behavior. Psychol. Inq. 11, 227–268. doi: 10.1207/S15327965PLI1104_01

[ref12] Dempster A. P. Laird N. M. Rubin D. B. (1977). Maximum likelihood from incomplete data via the EM algorithm. J. R. Stat. 39, 1–22. doi: 10.1111/j.2517-6161.1977.tb01600.x

[ref13] Digital Transformation Office of Turkey (2021). National Artificial Intelligence Strategy 2021–2025. Ankara, Türkiye: Presidency of the Republic of Turkey.

[ref14] El-Sayed A. A. I. Alsenany S. A. Asal M. G. R. Alasqah I. (2025). Development and validation of artificial intelligence addiction scale for researchers: a methodological study. J. Nurs. Manag. 2025:8458533. doi: 10.1155/jonm/8458533, 41424990 PMC12714078

[ref15] Epskamp S. (2015). SemPlot: unified visualizations of structural equation models. Struct. Equ. Model. 22, 474–483. doi: 10.1080/10705511.2014.937847

[ref16] Gerlich M. (2025). AI tools in society: impacts on cognitive offloading and the future of critical thinking. Societies 15:6. doi: 10.3390/soc15010006

[ref17] Giray L. (2024). Negative effects of generative AI on researchers: publishing addiction, dunning-Kruger effect and skill erosion. JALT 7, 398–405. doi: 10.37074/jalt.2024.7.2.38

[ref18] Goh A. Y. H. Hartanto A. Majeed N. M. (2025). Generative artificial intelligence dependency: scale development, validation, and its motivational, behavioral, and psychological correlates. Comput. Hum. Behav. Rep. 20:100845. doi: 10.1016/j.chbr.2025.100845

[ref19] Goodman A. (1990). Addiction: definition and implications. Br. J. Addict. 85, 1403–1408. doi: 10.1111/j.1360-0443.1990.tb01620.x, 2285834

[ref20] Griffiths M. D. (2005). A “components” model of addiction within a biopsychosocial framework. J. Subst. Use 10, 191–197. doi: 10.1080/14659890500114359

[ref21] Hair J. F. Black W. C. Babin B. J. Anderson R. E. (2022b). Multivariate data analysis. 8th Edn Cengage.

[ref22] Hair J. F. Hult G. T. M. Ringle C. M. Sarstedt M. (2022a). A primer on Partial least Squares Structural Equation Modeling (PLS-SEM). 3rd Edn Thousand Oaks, CA, USA: Sage.

[ref23] Hambleton R. K. Patsula L. (1998). Adapting tests for use in multiple languages and cultures. Soc. Indic. Res. 45, 153–171. doi: 10.1023/a:1006941729637

[ref24] Hao X. Demir E. Eyers D. (2024). Exploring collaborative decision-making: a quasi-experimental study of human and generative AI interaction. Technol. Soc. 78:102662. doi: 10.1016/j.techsoc.2024.102662

[ref25] Hu L.-T. Bentler P. M. (1999). Cutoff criteria for fit indexes in covariance structure analysis: conventional criteria versus new alternatives. Struct. Equ. Model. 6, 1–55. doi: 10.1080/10705519909540118

[ref26] International Test Commission (2017). The ITC Guidelines for Translating and Adapting Tests. 2nd Edn.

[ref27] Kline P. (1986). A Handbook of test Construction. London, United Kingdom: Methuen.

[ref28] Kline R. B. (2023). Principles and Practice of Structural Equation Modeling. (5th ed.) New York, NY: Guilford Press.

[ref29] Klingbeil A. Grützner C. Schreck P. (2024). Trust and reliance on AI—an experimental study on the extent and costs of overreliance on AI. Comput. Hum. Behav. 160:108352. doi: 10.1016/j.chb.2024.108352

[ref30] Krejcie R. V. Morgan D. W. (1970). Determining sample size for research activities. Educ. Psychol. Meas. 30, 607–610. doi: 10.1177/001316447003000308

[ref31] Kwon M. Lee J.-Y. Won W.-Y. Park J.-W. Min J.-A. Hahn C. . (2013). Development and validation of a smartphone addiction scale (SAS). PLoS One 8:e56936. doi: 10.1371/journal.pone.0056936, 23468893 PMC3584150

[ref32] Limayem M. Hirt S. G. Cheung C. M. K. (2007). How habit limits the predictive power of intention: the case of information systems continuance. MIS Q. 31, 705–737. doi: 10.2307/25148817

[ref33] Raykov T. Gabler S. Dimitrov D. M. (2016). Maximal reliability and composite reliability: examining their difference for multicomponent measuring instruments using latent variable modeling. Struct. Equ. Model. 23, 384–391. doi: 10.1080/10705511.2014.966369

[ref34] Revelle W. (2015). psych: Procedures for Personality and Psychological Research (R Package Version 1.5.8). Evanston, IL: Northwestern University.

[ref35] Risko E. F. Gilbert S. J. (2016). Cognitive offloading. Trends Cogn. Sci. 20, 676–688. doi: 10.1016/j.tics.2016.07.002, 27542527

[ref36] Rosseel Y. (2012). Lavaan: an R package for structural equation modeling. J. Stat. Softw. 48, 1–36. doi: 10.18637/jss.v048.i02

[ref37] Salam M. Farooq M. S. Ikram A. Shahzad M. Ali A. Jaafar N. (2026b). Revised artificial intelligence device use acceptance (RAIDUA) model: exploring privacy concerns for socially responsible AI deployment and ethical AI leadership. J. Hosp. Tour. Insights 9, 496–518. doi: 10.1108/JHTI-02-2025-0322

[ref38] Salam M. Radović-Marković M. Farooq M. S. Marković D. Vučeković M. (2026a). Artificial intelligence driven digital transformation: mediating the relationship between artificial intelligence readiness and business process efficiency. IPSI Transactions on Internet Research 22, 32–41. doi: 10.58245/ipsi.tir.2602.04

[ref39] Schepman A. Rodway P. (2023). The general attitudes towards artificial intelligence scale (GAAIS): confirmatory validation and associations with personality, corporate distrust, and general trust. Int. J. Hum.-Comput. Interact. 39, 2724–2741. doi: 10.1080/10447318.2022.2085400

[ref40] Tabachnick B. G. Fidell L. S. (2013). Using Multivariate Statistics. 6th Edn Boston, MA: Pearson.

[ref41] TurkStat (2025). Artificial Intelligence Statistics, 2025. Ankara, Türkiye: Turkish Statistical Institute.

[ref42] Young K. S. (1998). Internet addiction: the emergence of a new clinical disorder. Cyberpsychol. Behav. 1, 237–244. doi: 10.1089/cpb.1998.1.237

