# Supplementary material for: Adaptation and validation of the generative AI dependency scale: evidence from construct validity, reliability and measurement invariance
Source: Front Psychol. 2026 Aug 6;17:1908473. doi: 10.3389/fpsyg.2026.1908473 (PMC13491653; doi:10.3389/fpsyg.2026.1908473)
Supplement: Supplementary file 1 [file Supplementary_file_1.docx]

**Appendix – 1.**

**Generative AI Dependency Scale / Üretken Yapay Zekâ Bağımlılığı Ölçeği**

*Türkçe ve İngilizce madde listesi* /*List of item in Turkish and English*

| **No** | **Türkçe** | **English** |
| --- | --- | --- |
| **Bilişsel meşguliyet  Cognitive Preoccupation** | | |
| 1 | Kararlarım çoğu zaman üretken yapay zekâdan etkilenir. | My decisions are often influenced by generative AI. |
| 2 | Gerekli olmasa bile üretken yapay zekâyı kullanma dürtüsü hissediyorum. | I feel an urge to use generative AI, even when it may not be necessary. |
| 3 | Elimdeki işle ilgisi olmasa bile üretken yapay zekâyı kullanmayı sabırsızlıkla bekliyorum. | I look forward to using generative AI, even if it is unrelated to the task at hand. |
| **Olumsuz sonuçlar Negative Consequences** | | |
| 4 | Üretken yapay zekâ kullanımı benim için endişe kaynağı oldu. | My use of generative AI has caused concerns for me. |
| 5 | Üretken yapay zekâ olmadan işlerimi veya diğer sorumlulukları tamamlamakta zorlanıyorum. | I have trouble completing work or other responsibilities without generative AI. |
| 6 | Üretken yapay zekâ olmadan yeteneklerime daha az güveniyorum. | I feel less confident in my abilities without generative AI. |
| 7 | Üretken yapay zekâ kullanmam, problem çözme becerilerimi veya verimliliğimi olumsuz etkiledi. | My use of generative AI has negatively affected my problem-solving skills or efficiency. |
| **Yoksunluk Withdrawal** | | |
| 8 | Üretken yapay zekâyı kullanamadığımda kendimi huzursuz hissediyorum. | I experience restlessness if I am unable to use generative AI. |
| 9 | Üretken yapay zekâyı kullanamadığımda kendimi tedirgin veya dikkati dağılmış hissediyorum. | I feel unsettled or distracted when I cannot use generative AI. |
| 10 | Üretken yapay zekâya erişemediğimde kopukluk hissi yaşıyorum. | I feel a sense of disconnection when I cannot access generative AI. |
| 11 | Üretken yapay zekâyı kullanamadığımda sinirleniyorum veya huysuzlanıyorum. | I get frustrated or irritable when I am unable to use generative AI. |

**Yanıt seçenekleri / Response options**

| **Türkçe** | **English** |
| --- | --- |
| 1 = Kesinlikle katılmıyorum | 1 = Strongly disagree |
| 2 = Katılmıyorum | 2 = Disagree |
| 3 = Kararsızım | 3 = Neither agree nor disagree |
| 4 = Katılıyorum | 4 = Agree |
| 5 = Kesinlikle katılıyorum | 5 = Strongly agree |

***Note 1.*** The Generative AI Dependency Scale consists of a total of 11 items and includes three subdimensions: Cognitive Preoccupation (3 items), Negative Consequences (4 items), and Withdrawal (4 items). The Cognitive Preoccupation dimension includes items reflecting individuals’ persistent mental focus on and urge to use generative AI systems. The Negative Consequences dimension assesses the adverse effects of excessive use on individuals’ performance and self-efficacy. The Withdrawal dimension measures feelings of discomfort and distress experienced when access to generative AI systems is restricted. All items are rated on a five-point Likert-type scale ranging from 1 (Strongly Disagree) to 5 (Strongly Agree). The items of the scale are presented in the Appendix.

***Note 2.*** The scale scores can be calculated both at the subdimension level and as a total score. Subscale scores for Cognitive Preoccupation, Negative Consequences, and Withdrawal are obtained by summing the items within each respective dimension. In addition, based on the results of the second-order confirmatory factor analysis, an overall Generative AI Dependency score can be computed by summing all items. Since all items are scored in the same direction, no reverse coding is required. Higher scores indicate higher levels of dependency on generative AI systems, whereas lower scores reflect lower levels of dependency.
